# Extensively Drug-Resistant Salmonella typhi Infection: From Pill to Scalpel

**DOI:** 10.7759/cureus.25840

**Published:** 2022-06-10

**Authors:** Fawad Rahim, Said Amin, Mohammad Noor, Khushal Nadir Hadi, Sana Aftab

**Affiliations:** 1 Internal Medicine, Khyber Girls Medical College, Peshawar, PAK; 2 Internal Medicine, Hayatabad Medical Complex Peshawar, Peshawar, PAK

**Keywords:** abdominal pain, appendectomy, acute abdomen, appendicitis, extensively drug resistant typhoid, extensively drug resistant (xdr), salmonella enterica serovar typhi

## Abstract

Water-borne infections like typhoid fever are common in the developing world. The emergence of extensively drug-resistant Salmonella typhi (XDR S. typhi) is of great concern for both local and global public health. Fever, diarrhea, and abdominal pain are the commonest manifestations of typhoid fever. Abdominal pain may be due to ileal and colonic inflammation/ulceration and mesenteric lymphadenitis. Sometimes, abdominal pain in typhoid is due to ileal perforation leading to peritonitis, and acute appendicitis which needs urgent surgical intervention. Delayed surgical intervention can result in morbidity and sometimes even death.

We report a case of XDR S. typhi infection in a 17-year-old female who presented with fever and abdominal pain. During the course of the hospital stay, while she was on appropriate antibiotics, her abdominal pain worsened due to acute appendicitis. She underwent an appendectomy and had an uneventful recovery. This is the first case, to our knowledge, of acute appendicitis caused by XDR S. typhi.

Although appropriate antibiotics are the mainstay of treatment for typhoid fever, physicians should be mindful that surgical consultation may be necessary in certain cases.

## Introduction

In developing countries, where there is a lack of proper sewage disposal and clean drinking water, waterborne illnesses like cholera, typhoid fever, and viral hepatitis are still significant public health concerns [1]. Globally, up to 20 million people get infected with Salmonella typhi (S. typhi), resulting in 128,000 to 160,000 deaths every year [2].

Fever, abdominal pain, and diarrhea are the most common manifestations of typhoid fever. Abdominal pain in typhoid fever is multifactorial. It may be due to inflamed ileum, perforation of ileum leading to peritonitis, ileocolonic inflammation, mesenteric lymphadenitis, or acute appendicitis. Rarely, typhoid fever is complicated by gastrointestinal bleeding, visceral abscesses, vertebral abscess, osteomyelitis, and encephalopathy [3].

Acute appendicitis is a common surgical emergency, but it is rare to get acute appendicitis due to S. typhi. Cases of acute appendicitis due to drug-sensitive S. typhi in children and adults have been reported in the past [4-5]. With its emergence in 2018 in Hyderabad, Pakistan, 14,360 cases of extensively drug-resistant Salmonella typhi (XDR S. typhi) have been reported by the National Institute of Health (NIH), Pakistan [6]. The outbreak is not only a local health issue but cases of XDR S. typhi have been reported in the United States of America (USA), the United Kingdom, and Canada among travelers from Pakistan [7]. Nine cases of XDR S. typhi have been reported in the USA among those who did not travel outside the USA [8]. The outbreak is threatening global health as an XDR S. typhi strain resistant to azithromycin has already been reported in Bangladesh [9].

Acute hepatitis, visceral abscesses in organs like the spleen and splenic and renal infarcts have been reported in association with XDR S. typhi [10-11]. During the course of our literature search, this is the first case report of acute appendicitis caused by XDR S. typhi.

## Case presentation

A 17-year-old girl presented to the emergency room of Hayatabad Medical Complex, Peshawar, Pakistan, with complaints of fever, loose stools, and abdominal pain for eight days. The fever was continuous, high-grade, spiking up to 104 ^0^F. She also had watery diarrhea, about eight episodes per day, associated with abdominal pain. The pain was dull in nature, localized to the epigastrium and the right hypochondrium, with radiation to the right flank and right iliac fossa. She was diagnosed as having type 1 diabetes mellitus three years ago. She was on a basal-bolus insulin regimen with variable glycemic control. She used to take meals from nearby restaurants where food hygiene was substandard. None of her family members had similar symptoms.

On physical examination, she had a temperature of 102 ^0^F, blood pressure of 100/60 mmHg, pulse of 105 beats per minute, and oxygen saturation of 98% on room air. She was tender in the right upper abdomen and bowel sounds were present. The rest of the physical examination was unremarkable.

She was admitted to the medical unit for a workup. Food poisoning, enteric fever, amebiasis, acute viral hepatitis, and shigellosis were considered in the differential diagnosis. Blood culture, stool routine examination, and ultrasound of abdomen and pelvis were requested in addition to routine investigations. She was started on meropenem as empiric treatment for enteric fever and fluid replacement. The investigations are summarized in Table 1.

**Table 1:** Investigations at the time of admission g/dl: Gram per deciliter, mcL: Microliter, mg/dL: Milligram per deciliter, PT: Prothrombin time, APTT: Activated partial thromboplastin Time, IU/L: International unit per liter, g/dL: Gram per deciliter, ELISA: Enzyme-linked immunosorbent assay, HBsAg: Hepatitis B surface antigen, HCV: Hepatitis C virus, HIV: Human immunodeficiency virus, PCR: Polymerase chain reaction, NS: Non-structural protein, SARS-CoV-2: Severe acute respiratory syndrome coronavirus 2, IgM: Immunoglobulin M, IgG: Immunoglobulin G

Investigations	Reference range	Results
Hemoglobin (g/dL)	13.5 – 17.5	10.4
Platelet count (x10^3^/mcL)	150 – 450	220
White cell count (x10^3^/mcL)	4.5 – 11	6.42
Neutrophils (%)	40 – 60%	64
Lymphocytes (%)	20 – 40%	33
Monocytes (%)	2 – 8%	03
Eosinophils (%)	1 – 4%	00
C-Reactive Protein (mg/dL)	< 0.5	33.5
Total Bilirubin (mg/dL)	0.2 – 1.2	0.5
Alanine Aminotransferase (IU/L)	< 45	82
Alkaline Phosphatase (IU/L)	< 350	333
Serum Albumin (g/dL)	3.4 – 5.5	3.5
Serum Creatinine (mg/dL)	0.5 – 1.2	0.5
Urea (mg/dL)	20 – 40	13
PT (seconds)	12	12
APTT (seconds)	28	35
HBsAg (ELISA)	Non-reactive	
Anti-HCV (ELISA)	Non-reactive	
Anti-HIV (ELISA)	Non-reactive	
Dengue NS-1 antigen	Non-reactive	
Dengue IgM Antibodies	Non-reactive	
SARS-CoV-2 PCR	Not detected	
Malarial Parasite	Not seen	
Stool Analysis	Within normal limits	
Urine Analysis	Within normal limits	
Ultrasound of Abdomen and Pelvis	Few mesenteric lymph nodes, the largest measuring up to 8 mm	

**Figure 1:**
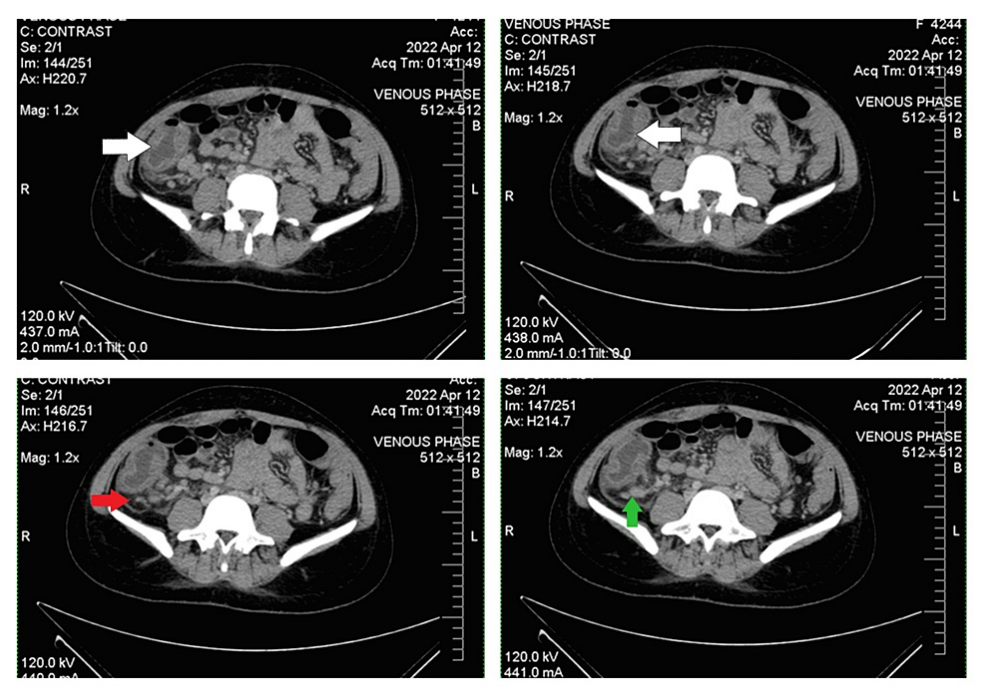
Contrast-enhanced computed tomography of the abdomen and pelvis showing thick-walled, inflamed appendix and cecum (white arrows), surrounding fat stranding (red arrow), and mesenteric lymphadenopathy (green arrow)

On day three of her admission, the abdominal pain increased in intensity, with severe tenderness in the right iliac fossa and right flank region. The surgical team advised contrast-enhanced computed tomography of the abdomen and pelvis. It revealed signs of a thick-walled appendix with free intraperitoneal fluid and mesenteric lymphadenopathy suggestive of acute appendicitis without definite evidence of perforation (Figure 1).

**Figure 2:**
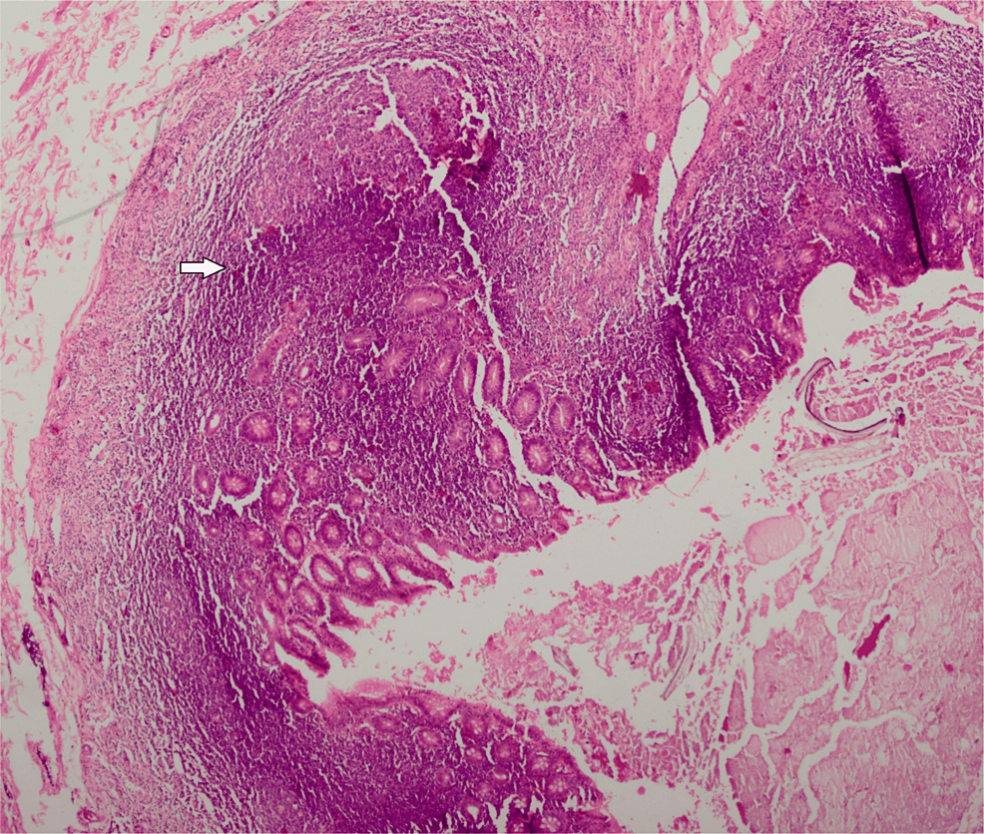
Low power view of the wall of the appendix showing acute inflammation

**Figure 3:**
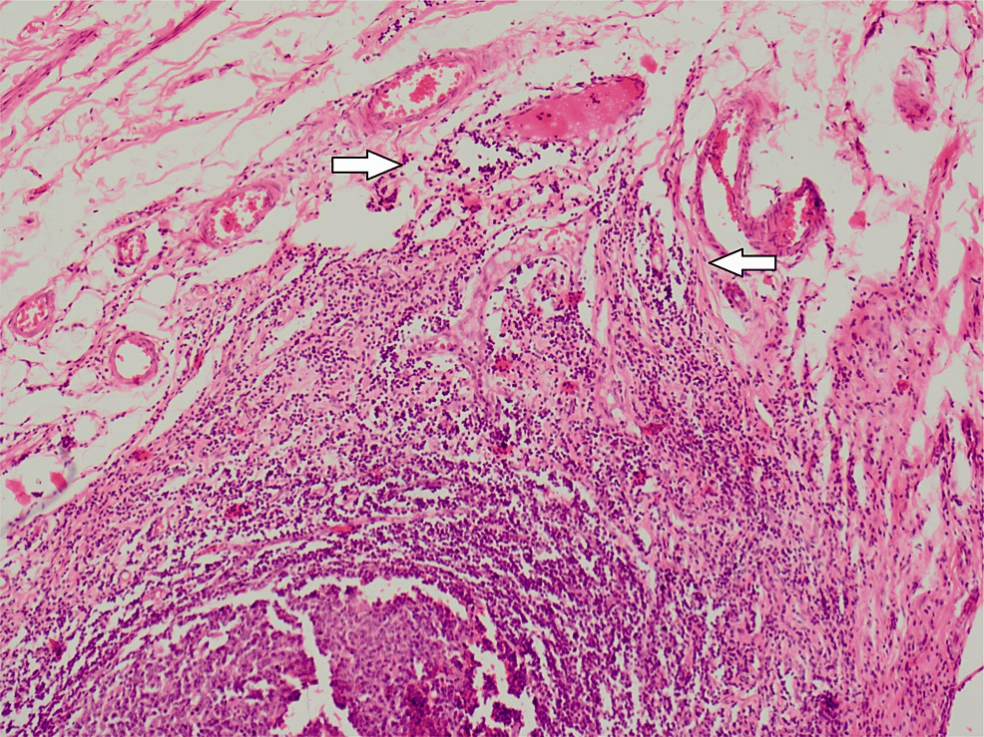
Inflammatory infiltrate in the peri-appendicular tissues, disrupting the muscular layer

**Figure 4:**
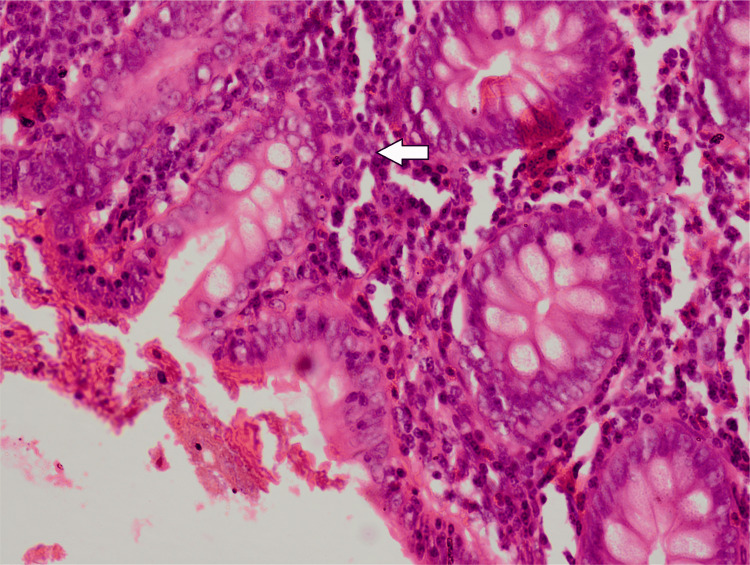
Medium power view of the appendix showing mucosal and submucosal inflammation

She underwent an emergent appendectomy. During surgery, 300 ml of reactive straw-colored fluid was drained, and a phlegmonous appendix was excised and sent for histopathological analysis. Meanwhile, the preliminary report of her blood culture suggested the growth of gram-negative rods, which was highly suggestive of S. typhi. She became pain-free after the surgery, but the fever persisted for the next two days. The final report of her blood culture demonstrated growth of XDR S. typhi sensitive only to imipenem, meropenem, and azithromycin, while resistant to ampicillin, fluoroquinolones, ceftriaxone, cefixime, co-trimoxazole, and chloramphenicol. She remained pain-free and afebrile and was discharged on the seventh postoperative day. She received meropenem for a further one week as an outpatient. She was asymptomatic during a follow-up visit in the outpatient department two weeks after discharge. The histopathology report was suggestive of acute appendicitis (Figures 2-4).

## Discussion

The surgical complications of typhoid fever cause significant morbidity and mortality in many parts of Africa and Asia where standard medical facilities are not yet readily available [12]. The gastrointestinal manifestations of enteric fever include gastrointestinal ulcers leading to hemorrhage and perforation. Additionally, acute cholecystitis, acute pancreatitis, acute gastroenteritis, acute appendicitis, and post-infectious irritable bowel syndrome have been reported [10,13].

Acute appendicitis is one of the most common surgical emergencies and is caused by luminal obstruction of the appendix by fecolith, tumor, or submucosal lymphoid hyperplasia with superadded infection [14]. S. typhi can cause acute abdomen mimicking acute surgical emergencies, and it is difficult to ascertain the exact diagnosis, resulting in delayed surgical intervention and an increase in morbidity. Around 8% of cases of acute appendicitis are due to drug-sensitive S. typhi [15]. Cases of acute appendicitis caused by drug-sensitive S. typhi have been reported by Zheng et al. from China, Sartori from the USA, and Lau Susanna et al. from Hong Kong [4,15-16]. Histologically, it is similar to nonspecific appendicitis characterized by mucosal and submucosal lymphoid hyperplasia, neutrophilic infiltration, and abscess formation in the appendix [16].

We report a unique case of a young girl with type 1 diabetes mellitus and acute appendicitis caused by XDR S. typhi. Gastrointestinal and hepatobiliary involvement in XDR S. typhi infection has been reported. Abscesses in various viscera like the spleen and infarcts in the spleen and kidneys caused by XDR S. typhi have been reported [10-11]. Similarly, hepatic involvement manifested by more than a three-fold elevation of alanine aminotransferase in 14% of cases of XDR S. typhi has been reported [10]. During the course of our literature search, this is the first case of acute appendicitis caused by XDR S typhi. This case would add to the emerging data on the surgical complications of XDR S. typhi.

## Conclusions

Abdominal pain in XDR S. typhi infection is multifactorial. Some causes of abdominal pain like ileocolic inflammation and ulceration and mesenteric lymphadenitis settle with appropriate antibiotic therapy. But the physician should be vigilant for ileal perforation leading to peritonitis and acute appendicitis as a cause of abdominal pain that needs surgical intervention. This case highlights the importance of having a high index of suspicion for the surgical complications of XDR S. typhi infection. Timely intervention for these complications would reduce morbidity and mortality.
